# Chlorpromazine and Promethazine (C+P) Reduce Brain Injury after Ischemic Stroke through the PKC-*δ*/NOX/MnSOD Pathway

**DOI:** 10.1155/2022/6886752

**Published:** 2022-07-15

**Authors:** Sichao Guo, Fengwu Li, Melissa Wills, James Yip, Alexandra Wehbe, Changya Peng, Xiaokun Geng, Yuchuan Ding

**Affiliations:** ^1^Luhe Institute of Neuroscience, Beijing Luhe Hospital, Capital Medical University, Beijing 101100, China; ^2^Department of Neurosurgery, Wayne State University School of Medicine, Detroit, MI 48201, USA; ^3^Department of Research & Development Center, John D. Dingell VA Medical Center, Detroit, MI 48201, USA; ^4^Harvard T.H. Chan School of Public Health, Boston, MA 02115, USA; ^5^Department of Neurology, Beijing Luhe Hospital, Capital Medical University, Beijing 101100, China

## Abstract

Cerebral ischemia-reperfusion (I/R) incites neurologic damage through a myriad of complex pathophysiological mechanisms, most notably, inflammation and oxidative stress. In I/R injury, nicotinamide adenine dinucleotide phosphate (NADPH) oxidase (NOX) produces reactive oxygen species (ROS), which promote inflammatory and apoptotic pathways, augmenting ROS production and promoting cell death. Inhibiting ischemia-induced oxidative stress would be beneficial for reducing neuroinflammation and promoting neuronal cell survival. Studies have demonstrated that chlorpromazine and promethazine (C+P) induce neuroprotection. This study investigated how C+P minimizes oxidative stress triggered by ischemic injury. Adult male Sprague-Dawley rats were subject to middle cerebral artery occlusion (MCAO) and subsequent reperfusion. 8 mg/kg of C+P was injected into the rats when reperfusion was initiated. Neurologic damage was evaluated using infarct volumes, neurological deficit scoring, and TUNEL assays. NOX enzymatic activity, ROS production, protein expression of NOX subunits, manganese superoxide dismutase (MnSOD), and phosphorylation of PKC-*δ* were assessed. Neural SHSY5Y cells underwent oxygen-glucose deprivation (OGD) and subsequent reoxygenation and C+P treatment. We also evaluated ROS levels and NOX protein subunit expression, MnSOD, and p-PKC-*δ*/PKC-*δ*. Additionally, we measured PKC-*δ* membrane translocation and the level of interaction between NOX subunit (p47^phox^) and PKC-*δ* via coimmunoprecipitation. As hypothesized, treatment with C+P therapy decreased levels of neurologic damage. ROS production, NOX subunit expression, NOX activity, and p-PKC-*δ*/PKC-*δ* were all significantly decreased in subjects treated with C+P. C+P decreased membrane translocation of PKC-*δ* and lowered the level of interaction between p47^phox^ and PKC-*δ*. This study suggests that C+P induces neuroprotective effects in ischemic stroke through inhibiting oxidative stress. Our findings also indicate that PKC-*δ*, NOX, and MnSOD are vital regulators of oxidative processes, suggesting that C+P may serve as an antioxidant.

## 1. Introduction

Acute ischemic stroke results in permanent neurologic damage, contributing to its high morbidity and mortality rates internationally and high health care costs annually [1]. Reperfusion strategies, such as tissue plasminogen activator (tPA) and thrombectomy, are the standard for treating acute ischemic stroke and have been the primary focus of technological advances in stroke therapy [2]. However, even in the event of successful recanalization, morbidity rates remain high and clinical outcomes are often poor due to complicating reperfusion injury [3, 4]. It is imperative to explore other therapeutic methods, such as neuroprotective and neuro-rehabilitative strategies, to maximize functional outcomes and patient quality of life.

Chlorpromazine and promethazine (C+P), two phenothiazine drugs, are commonly utilized for their sedative and antipsychotic effects [5]. C+P induces a state of “artificial hibernation,” an effect found to confer neuroprotection in cerebral ischemia [5–7]. Previous studies suggest that the hypothermia induced by this state of hibernation is key to its potent neuroprotective properties. Reduced body temperatures and metabolic rates confer a higher tolerance to ischemia, hypoglycemia, and other conditions of low perfusion states, thus presenting more favorable parameters for patients with acute ischemic stroke. Pharmacological hypothermia, induced by drugs such as C+P, evades many potential complications associated with therapeutic hypothermia induced through external or endovascular cooling methods, such as coagulopathy, hypokalemia, and infection [8]. However, some studies have shown that the neuroprotective mechanisms of C+P are independent or only partially dependent on temperature, thus suggesting that C+P's neuroprotective effects may be due to a separate, temperature-independent mechanism [5, 9]. Phenothiazines have been found to protect the *in vitro* human neuroblastoma cell line SHSY5Y from oxidative stress triggered by both exogenic and mitochondrial free radicals. Phenothiazines act as neuroprotectants by both scavenging free radicals and inducing antioxidant protein synthesis [10]. Furthermore, promethazine was shown to be protective in stroke models by targeting the mitochondrial permeability transmission (mPT), which, when activated, has been linked to cytotoxicity after infective, ischemic, and excitotoxic pathological insults [11].

Dual therapy with C+P has been found to have a significant protective effect on neurologic tissue in both transient and permanent ischemic strokes. C+P has been found to decrease infarction volumes, improve long-term motor performance, and minimize metabolic damage [5]. Prior studies have suggested that C+P may induce neuroprotection through changes in protein levels of PKC-*δ* and nicotinamide adenine dinucleotide phosphate oxidase (NOX) [5, 12], which are protein complexes heavily involved in oxidative stress and cell destruction after ischemic stroke. However, the specific mechanism by which C+P inhibits oxidative stress through the PKC-*δ*/NOX pathway is still unclear. To fully understand the neuroprotective effects of C+P, this study investigated mechanisms by which C+P inhibits oxidative stress after stroke.

Excess oxygen and reactive oxygen species (ROS) are widely known to produce oxidative stress, causing cellular damage after ischemic stroke [13]. NOX, a multi-subunit enzyme, employs nicotinamide adenine dinucleotide phosphate (NADP+) to generate ROS and superoxide anions [14]. Increases in NOX activity, NOX subunit expression, and cell death have been found after ischemia/reperfusion (I/R) and are associated with larger infarct volumes and worse functional outcomes [14–16]. NADPH oxidase is comprised mainly of p22^phox^ (phox, phagocyte oxidase), p47^phox^, and p67^phox^, which are regulatory subunits, and catalytic subunit gp91^phox^ [17]. The brain has been previously shown to express the major NOX subunits (p22^phox^, p47^phox^, p67^phox^, and gp91^phox^) [15] and activate NOX, which aggravates I/R injury by overproducing ROS [15]. Additionally, protein kinase C (PKC) acts as a key activator of NADPH oxidase [18]. As a member of the PKC family, PKC-*δ* activates NOX during I/R, augmenting oxidative damage and cell apoptosis [12]. Conversely, manganese-containing superoxide dismutase (MnSOD), a vital antioxidant enzyme in the central nervous system, is a main pathway through which cells resist ROS damage after cerebral ischemia [19].

Our study sought to delineate the antioxidation mechanism by which C+P induces neuroprotective effects by assessing the PKC-*δ*/NOX/MnSOD pathway, as well as the capacity of these drugs to repair oxidative damage after ischemic stroke. A robust understanding of the oxidative pathway of neuroprotection after ischemic stroke would guide future endeavors in finding therapeutic targets that capitalize on these processes to improve patient morbidity and mortality.

## 2. Materials and Methods

### 2.1. Subjects

142 adult male Sprague-Dawley rats (280–300 g, Beijing Vital River Laboratory Animal Technology Company, Limited) served as the subjects of our study. We followed the Institutional Animal Investigation Committee of the Capital Medical University standard operating procedures (89E58F61-7D97-4FA1-B7D0-02DA47F7A735). The animals were caged and subjected to a 12-hour light/dark cycle for the duration of the experiment. All possible steps were taken to minimize harm to the rats and the quantity of rats used. The rats were separated into six groups randomly: (1) a sham group that did not undergo middle cerebral artery occlusion (MCAO) (*n* = 12); (2) two-hour MCAO before reperfusion for 6 or 24 hours (*n* = 26); (3) two-hour MCAO and C+P with temperature control at 37°C before reperfusion for 6 or 24 hours (*n* = 26); (4) two-hour MCAO and C+P without temperature control before reperfusion for 6 or 24 hours (*n* = 26); (5) two-hour MCAO and NOX inhibitor before reperfusion for 6 or 24 hours (*n* = 21); and (6) two-hour MCAO and PKC-*δ* inhibitor before reperfusion for 6 or 24 hours (*n* = 21). Ten rats (<10%) were not included in further analysis due to either cerebral hemorrhage, death, or absence of ischemic injury based on neurological deficit scoring and TTC staining. The experiment and statistical analysis were conducted in a random and blinded fashion [20]. For the temperature-controlled groups, rats were put under warm ambient light on 37°C insulation blankets to maintain body temperature. For the non-temperature-controlled groups, animals were stored in a 25°C facility. Due to the intimate linkage between cerebral and body temperatures [21], rectal temperatures were monitored to lower the risk of intracranial hemorrhage.

### 2.2. Focal Cerebral Ischemia

We have previously explained the model of regional cerebral ischemia employed for this study [22]. In summary, the subjects were placed within a chamber and anesthetized with 1–3% isoflurane and a 30% oxygen and 70% nitrous oxide solution. Using a face mask, anesthesia was with 1% isoflurane administered via a calibrated precision vaporizer. To minimize variability between rodents and yield consistently sized infarcts, the MCA was occluded using 4.0 poly-L-lysine-coated intraluminal nylon sutures for each procedure.

### 2.3. Chlorpromazine+Promethazine Administration and NOX Inhibitor/PKC-*δ* Inhibitor

In equal parts, rats received 8 mg/kg of C+P intraperitoneally (IP) when reperfusion began, the parameters of which were validated in a prior study [5]. One to two hours later, rats were given another injection, which was dosed at one-third of the original dose of C+P, to augment the C+P's therapeutic effects. Either a NOX inhibitor, apocynin (2.5 mg/kg), or a PKC-*δ* inhibitor, rottlerin (0.3 mg/kg), was injected IP 2 hours from initiation of MCAO.

### 2.4. Cell Culture and Oxygen Glucose Deprivation/Reoxygenation (OGD/R) with C+P Administration

The cell line of human SHSY5Y was acquired from ATCC. We incubated cells in a 25 cm^2^ culture flask with 5 ml of DMEM/F12 and 10% FBS and cultured to nine to eleven passages. We incubated the cells at 5% CO_2_ and 37°C in a humidified incubator, explained in a previous study by us [23].

To execute the OGD model, described in a previous study by us, an anaerobic chamber flushed with 95% N_2_ and 5% CO_2_ (*v*/*v*) maintained at 37°C was used [23]. We replaced culture medium with deoxygenated and glucose-free DMEM to begin two hours of OGD. The SHSY5Y cells were taken out of the anaerobic chamber, and the DMEM medium was exchanged with maintenance medium, with subsequent C+P treatment. C+P had a final concentration of 2.5 *μ*M, determined in a prior project conducted by us. Lastly, in a humidified incubator, cells were cultured for either 6 or 24 hours before harvest.

### 2.5. Infarct Volume Measurement

After being removed from the cranial cavity, the brain matrix was cut into 2 mm thick slices and stained using 2,3,5-triphenyltetrazolium chloride after reperfusion for 48 hours. Infarction volumes were calculated indirectly to minimalize error caused by edema. Cross-sectional areas of the infarcted regions in each slice were computed with ImageJ. Infarction volume for each slice was computed as the difference between total left hemisphere volume and volume of the noninfarcted right hemisphere, then divided by the total volume of the left hemisphere [24].

### 2.6. TUNEL Assay

The TUNEL assay was employed for evaluating DNA fragmentation using a commercial kit as outlined in prior work conducted by us [24, 25]. Images were obtained randomly from territory supplied by the MCA. Complying with the manufacturer's indications, the permeabilized and fixed slides were incubated in 50 *μ*l of TUNEL reaction mixture for one hour at 37°C. Positive TUNEL staining was visualized with fluorescent microscopy. The TUNEL^+^ cells were counted using the ImageJ manual cell counting tool. The cell counts from every section from each group for the statistical analysis were blindly obtained. The total cell number was acquired via counting the DAPI^+^ cells, including each dead cell. From each rat, a slide was selected (*n* = 5), and four regions of interest were assessed from each slide. The percent of apoptotic cells was computed and averaged as the (total TUNEL^+^ cells in all 20 regions/total cells in all 20 regions) × 100%. Because the images were acquired randomly from four separate areas in MCA-supplied territory, findings signify cell death of the area of ischemia.

### 2.7. Cell Death Detection

Apoptotic cell death was assessed by a photometric enzyme immunoassay explained in a previous study conducted by us [26]. The number of cytoplasmic histone-associated DNA fragments created through apoptotic cell death was assessed. Subsequently, light absorbance at a 405 nm wavelength was computed.

### 2.8. NADPH Oxidase Activity

Right cerebral hemisphere sample tissues were processed using methods explained in our prior study [27]. In summary, cerebral tissue samples were homogenized with lysis buffer. In each well of the luminescence plate, 80 *μ*l lucigenin mix and 20 *μ*l of homogenate were added and subsequently incubated at 37°C for 10 minutes. The final concentration of NADPH, 0.1 mM, was then added, and we determined luminescence using the DTX-880 multimode detector for thirty seconds per specimen, totaling at ten minutes.

### 2.9. ROS Assay

Neurologic tissue samples comprised of MCA-supplied territories, the frontoparietal cortex, and striatum were analyzed. The Amplex Red Hydrogen Peroxide/Peroxidase Assay Kit was used to identify ROS, which we described in a prior study [28]. In the brain homogenates, the amount of H_2_O_2_ was measured with a DTX-880 Multimode Detector.

To measure ROS production in cells, we utilized DCFH-DA (2,7-dichlorodihydrofluorescein diacetate), which we explained in a prior study [23]. Cultures were washed one time using PBS and then stained with 25 *μ*M DCFDA in 100 *μ*l PBS at 37°C for a half hour. Cultures were washed thrice with PBS prior to signal reading at Ex/Em 485/535 nm.

### 2.10. Coimmunoprecipitation (Co-IP)

Coimmunoprecipitation of PKC-*δ* with p47^phox^ was performed. Cell supernatants were collected 6 and 24 hours after OGD/R. Cell lysis buffer (9803, CST) was used to collect the lysates. For 15 minutes and at 4°C, the lysates were centrifuged at 13,000 rpm prior to collecting the supernatants. Primary anti-PKC-*δ* (sc-213, Santa Cruz Biotechnology) and anti-IgG antibody (2729, Cell Signaling Technology) were added to 200 *μ*l cell lysate and incubated at 4°C overnight. Antibody immunocomplex solution and lysate were transferred to the protein G magnetic bead-containing tube and incubated with rotation at room temperature for 20 minutes. Magnetic Separation Rack (7017, Cell Signaling Technology) was used to separate the magnetic beads in accordance with the manufacturing directions and then boiled to denature the protein-bead complex. The immunoprecipitants from cells underwent Western blot analysis. Primary anti-PKC-*δ* (9616, Cell Signaling Technology) and anti-p47^phox^ (4312, Cell Signaling Technology) were used for Western blot detection.

### 2.11. Cellular Subfraction Analysis

The subcellular protein fractionation kit for cultured cells was used to execute the subcellular fractionation in accordance with manufacturing directions [29]. Whole cell lysates which were cultured to confluence were prepared using lysis buffer that included protease inhibitors. Proteins were then separated by SDS PAGE. The primary antibodies used were anti-PKC-*δ* and Na^+^/K^+^ ATPase as indicators of membrane subfraction, whereas *α*-tubulin was used as the indicator for the cytoplasm subfraction.

### 2.12. Protein Expression

Protein expression in brain tissue and cells was detected using Western blot analysis, which we have described in a prior study [28]. The frontoparietal cortex and striatum (right cerebral hemisphere components) were managed as the tissue sample for subsequent evaluation. A mammalian protein extraction reagent was used to lyse tissues and cells. Isolated rodent brain protein and cell isolates were then loaded onto electrophoresis gels. Then, proteins were moved to a polyvinylidene fluoride membrane. Membranes were then incubated with primary antibodies (1 : 1,000, rabbit anti-PKC-*δ* antibody; 1 : 1,000, anti-p-PKC-*δ* antibody; 1 : 1,000, rabbit anti-p47^phox^; 1 : 1,000, rabbit anti-p22^phox^; 1 : 1,000, rabbit anti-gp91^phox^; 1 : 1,000, rabbit anti-p67^phox^; 1 : 1,000, anti-MnSOD antibody; and 1 : 2,000, *β*-actin) for 16 hours at 4°C. Subsequently, membranes were then incubated using a secondary antibody (goat anti-rabbit IgG) for 2 hours at room temperature. Immunoreactive bands were identified with an enhanced chemiluminescence system. For each antibody, Western blot images were studied with ImageJ to quantify relative image density of protein expression.

### 2.13. Statistical Analysis

GraphPad Prism v8.0 (San Diego, California, United States) was used to conduct the statistical analyses. Using one-way ANOVA, differences among groups were computed with a predetermined significance level of *p* < 0.05. Post hoc comparison among groups was done using the least significant difference (LSD) method. Data are presented as the mean ± SE.

## 3. Results

### 3.1. C+P Minimized Neurologic Injury Postischemia-Reperfusion

**Figure 1:**
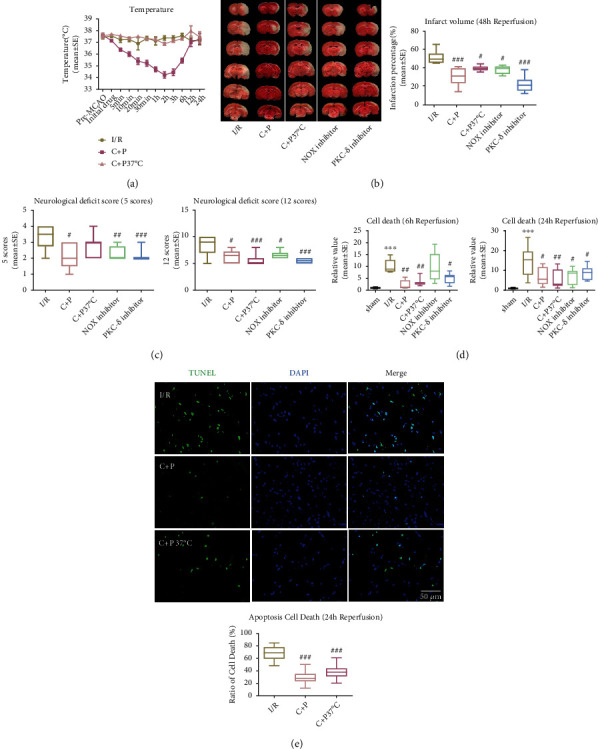
C+P lessened postischemic neurologic damage. (a) Alterations in body temperature post C+P administration at various points in time in 2-hour MCAO and after reperfusion (0–24 hours). Baseline temperatures were determined just before MCAO. Reperfusion was followed by C+P administration. ANOVA analyses specified that C+P significantly decreased body temperatures as early as 5 minutes and lasting up to 6 hours after the I/R onset (*n* = 7). (b) TTC stains showcase infarction volumes in MCAO rats regardless of C+P treatment at 48 hours of reperfusion. C+P administration or administration of the PKC-*δ*/NOX inhibitor significantly decreased infarct volumes (*n* = 7). (c) Neurologic deficits were determined using 5- or 12-point scoring systems at 48 hours of reperfusion. C+P administration or the administration of the PKC-*δ*/NOX inhibitor significantly reduced neurological deficits in all groups except those in the temperature-controlled C+P group, for which there was no effect on the 5-point scoring system (*n* = 7). (d) Cell death was significantly higher at 6 and 24 hours of reperfusion, demonstrated by ELISA. With C+P at 6 and 24 hours of reperfusion, cell death was significantly reduced. Administration of the PKC-*δ*/NOX inhibitor also suppressed cell death except in the NOX inhibitor group at 6 hours of reperfusion (*n* = 7). (e) Apoptotic cell death was significantly reduced with C+P administration regardless of temperature control at 24 hours of reperfusion, shown using the TUNEL assay (*n* = 5). Scale bar = 50 *μ*m. ^∗∗∗^*p* < 0.001 versus the sham group; ^#^*p* < 0.05, ^##^*p* < 0.01, and ^###^*p* < 0.001 versus the I/R group. Results are shown as mean ± SE.

Body temperature was significantly reduced as early as 5 minutes post-C+P treatment for the non-temperature-controlled C+P group versus the I/R or C+P at 37°C groups. Around 2 hours posttreatment, body temperatures were lowered to 34°C. Body temperatures continued to be significantly lowered for up to 6 hours, prior to spontaneously returning to normothermic levels (Figure 1(a)).

In comparison to the ischemic group with the greatest cerebral infarction volume at 48 hours of reperfusion (Figure 1(b)), infarct volumes were significantly reduced for both temperature-controlled and non-temperature-controlled C+P groups. Neurologic deficits were determined by previously validated 5- or 12-point scoring systems at 48 hours of reperfusion (Figure 1(c)). Compared with the I/R group, C+P administration in the non-temperature-controlled groups inhibited 5-point scoring system scores, indicating lower levels of neurological damage (*p* < 0.05). Similarly, the non-temperature-controlled C+P and C+P temperature-controlled groups (*p* < 0.05, *p* < 0.001) had lower 12-point scoring system scores, also indicating reduced neurologic damage. To examine the fundamental physiology behind C+P's neuroprotective effects in the context of the PKC-*δ*/NOX pathway post-stroke, we gave PKC-*δ* and NOX inhibitors. NOX and PKC-*δ* inhibition significantly reduced infarct volumes and neurological deficits at 48 hours of reperfusion (Figures 1(b) and 1(c)).

Apoptotic cell death assessed using ELISA was amplified post-MCAO as compared to the sham group but decreased after C+P administration at 6 and 24 hours after reperfusion (*p* < 0.05, *p* < 0.01) (Figure 1(d)). Additionally, the NOX and PKC-*δ* inhibitors significantly attenuated the higher levels of cell apoptosis, except for NOX inhibition groups after 6 hours of reperfusion (Figure 1(d)). Moreover, apoptotic cell death determined by the TUNEL assay was significantly reduced in groups who received C+P regardless of temperature control status (*p* < 0.001) (Figure 1(e)).

### 3.2. C+P Treatment Attenuated ROS Levels and NOX Activity Postreperfusion

**Figure 2:**
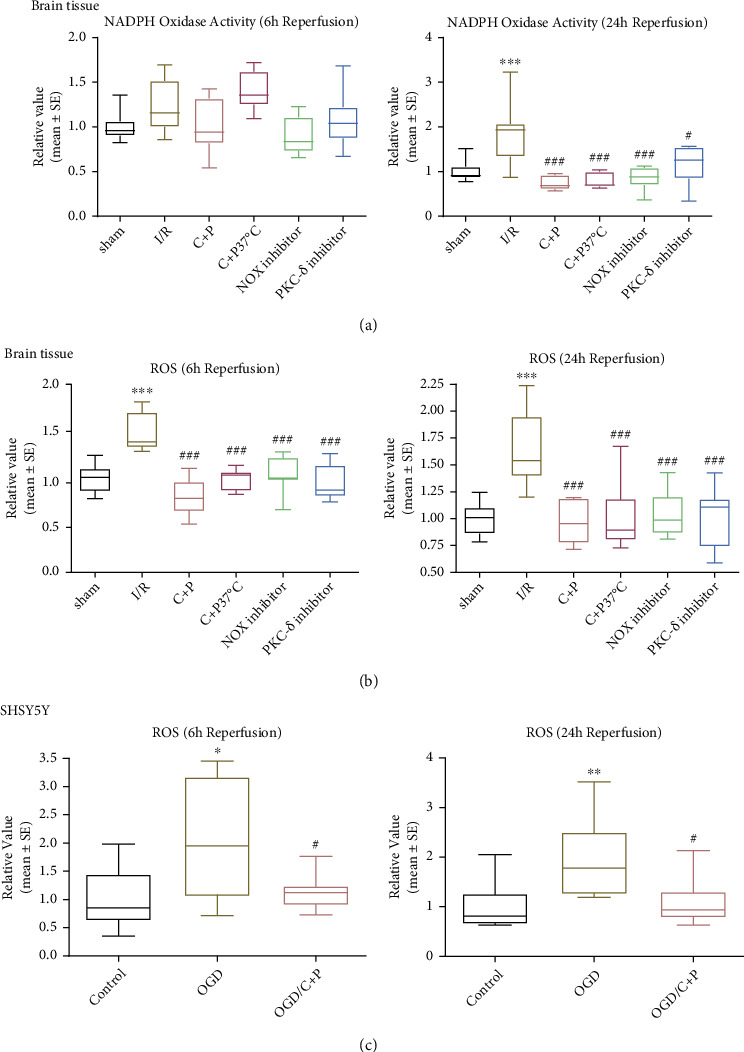
C+P attenuated NOX activity and ROS level postreperfusion. Quantitative analysis of (a) NOX activity and (b) ROS level as determined in ischemic brain tissue. NOX activity was higher at 24 hours of reperfusion, and ROS levels were also higher at 6 and 24 hours of reperfusion. Decreased NOX activity levels at 24 hours of reperfusion and ROS at 6 and 24 hours of reperfusion were observed in C+P groups regardless of temperature control as well as in PKC-*δ* or NOX inhibitor groups (*n* = 7). ^∗∗∗^*p* < 0.001 versus the sham group; ^#^*p* < 0.05, ^###^*p* < 0.001 versus the I/R group. Data is shown as the mean ± SE. Quantitative analysis of (c) ROS level in SHSY5Y cell was measured. C+P administration lowered the OGD-induced elevation of ROS at 6 and 24 hours of reoxygenation. ^∗^*p* < 0.05, ^∗∗^*p* < 0.01 versus the OGD group; ^#^*p* < 0.05 versus the OGD/C+P group. Results are shown as mean ± SE of three independent experiments.

In rats subject to 2 hours of MCAO, cerebral NOX activity was significantly higher at 24 hours of reperfusion. Adding C+P reduced NOX activity (*p* < 0.001). Moreover, NOX and PKC-*δ* inhibitors significantly lowered NOX activity at 24 hours of reperfusion (Figure 2(a)). We also found a significant surge in ROS generation at both 6 and 24 hours of reperfusion in neurologic tissue; however, C+P administration significantly reduced these ROS levels (Figure 2(b)). The NOX and PKC-*δ* inhibitors also reduced ROS at 6 and 24 hours of reperfusion (*p* < 0.001) (Figure 2(b)). Additionally, we examined C+P's influence on SHSY5Y cells with OGD/R. While ROS levels were raised at 6 and 24 hours of reoxygenation, C+P administration offered (*p* < 0.05) significant protection to the cells by inhibiting ROS production (Figure 2(c)).

### 3.3. C+P Treatment Decreased NOX Subunit Expression

**Figure 3:**
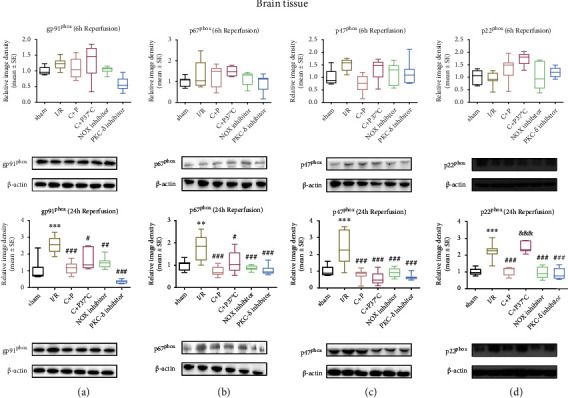
C+P decreased NOX subunit protein levels after stroke in ischemic brain tissue. (a) gp91^phox^, (b) p67^phox^, (c) p47^phox^, and (d) p22^phox^ assessed via the Western blot technique at 6 and 24 hours of reperfusion. Levels of gp91^phox^, p67^phox^, p47^phox^, and p22^phox^ rose after I/R but decreased after C+P treatment and administration of the PKC-*δ* or NOX inhibitor at 24 hours of reperfusion, with the exception of p22^phox^ in the C+P at the 37°C group at 24 hours of reperfusion. There was a larger decrease in p22^phox^ expression in the non-temperature-controlled C+P versus the temperature-controlled C+P group at 24 hours of reperfusion (*n* = 7). ^∗∗^*p* < 0.01 and ^∗∗∗^*p* < 0.001 versus the sham group; ^#^*p* < 0.05, ^##^*p* < 0.01, and ^###^*p* < 0.001 versus the I/R group; ^&&&^*p* < 0.001 versus the C+P group. Results are shown as mean ± SE. The representative immunoblots are visualized.

The ischemic group demonstrated significant enhancement of NOX subunit expression (gp91^phox^, p67^phox^, p47^phox^, and p22^phox^) at 24 hours of reperfusion (*p* < 0.01, *p* < 0.001). Both C+P therapy groups demonstrated decreased NOX subunit levels at 24 hours of reperfusion except p22^phox^ in the temperature-controlled C+P group (Figures 3(a)–3(d)). There was a larger decrease in p22^phox^ expression for non-temperature-controlled groups compared to temperature-controlled groups administered with C+P at 24 hours of reperfusion (*p* < 0.001) (Figure 3(d)). Also, NOX and PKC-*δ* inhibitors significantly reversed the raised NOX subunit levels at 24 hours of reperfusion (*p* < 0.01, *p* < 0.001) (Figures 3(a)–3(d)).

**Figure 4:**
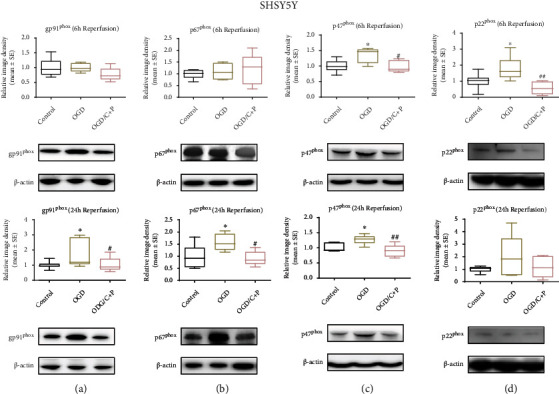
C+P reduced protein levels of NOX subunits after OGD/R in SHSY5Y cells. (a) gp91^phox^, (b) p67^phox^, (c) p47^phox^, and (d) p22^phox^, identified by Western blot at 6 and 24 hours of reoxygenation. Levels of gp91^phox^ and p67^phox^ protein increased after OGD but decreased after C+P therapy at 24 hours of reoxygenation. Moreover, p47^phox^ levels increased after OGD, but C+P lowered this expression at 6 and 24 hours of reoxygenation. OGD induced the elevation of p22^phox^, and there was a greater reduction in p22^phox^ expression in the OGD/C+P treatment group versus the OGD group at 6 hours of reoxygenation. ^∗^*p* < 0.05 versus the OGD group; ^#^*p* < 0.05, ^##^*p* < 0.01 versus the OGD/C+P group. Results are shown as mean ± SE of three independent experiments. The representative immunoblots are presented.

Also, in SHSY5Y cells with OGD/R treatment, levels of gp91^phox^ and p67^phox^ were enhanced at 24 hours of reoxygenation when assessed against the control group (*p* < 0.05); however, C+P significantly annulled raised gp91^phox^ and p67^phox^ protein levels (Figures 4(a) and 4(b)). OGD enhanced levels of p47^phox^ at 6 and 24 hours of reoxygenation (*p* < 0.05). Furthermore, C+P administration also lowered levels of p47^phox^ protein levels (*p* < 0.05, *p* < 0.01) at 6 and 24 hours of reoxygenation (Figure 4(c)). At 6 hours of reoxygenation, OGD-induced upregulation of p22^phox^ (*p* < 0.05) was negated by C+P treatment (*p* < 0.01) (Figure 4(d)).

### 3.4. C+P Treatment Suppressed Phosphorylation PKC-*δ* Activation

**Figure 5:**
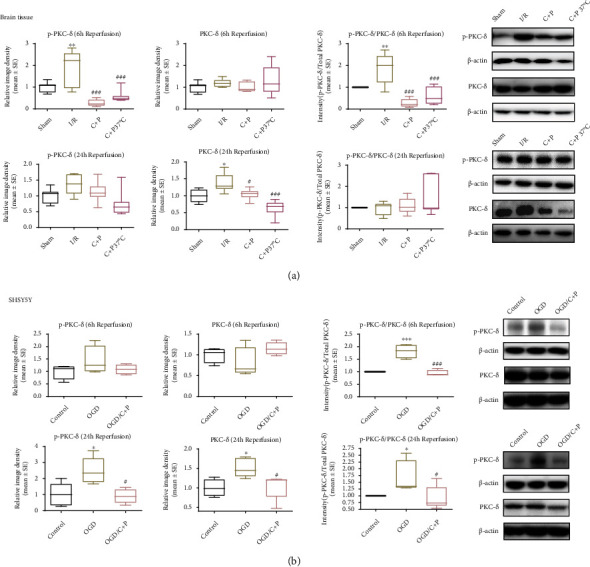
C+P decreased activation of PKC-*δ* induced by cerebral ischemia or OGD/R. (a) Representative bands of phosphorylated and total protein expressions of PKC-*δ* in brain tissue identified via Western blot at 6 and 24 hours of reperfusion. *β*-Actin served as an internal control. Bar graphs demonstrate semiquantitative levels of PKC-*δ*, identified using band density analysis. MCAO enhanced PKC-*δ* phosphorylation. C+P treatment reduced PKC-*δ* phosphorylation as compared with the stroke group at 6 hours of reperfusion. Total PKC-*δ* protein expression increased, but C+P reduced its level at 24 hours of reperfusion. Moreover, the ratio of p-PKC-*δ*/PKC-*δ* significantly increased post-MCAO but decreased with C+P treatment at 6 hours of reperfusion (*n* = 7). ^∗^*p* < 0.05, ^∗∗^*p* < 0.01 versus the sham group; ^#^*p* < 0.05, ^###^*p* < 0.001 versus the I/R group. Results are presented as mean ± SE. (b) Representative bands of both phosphorylated and total PKC-*δ* levels in SHSY5Y cells identified via Western blot at 6 and 24 hours of reoxygenation. *β*-Actin served as an internal control. Bar graphs display semiquantitative levels of PKC-*δ* as determined by band density analysis. The p-PKC-*δ*/PKC-*δ* ratio was significantly higher after OGD/R; however, it decreased after C+P administration at 6 hours of reoxygenation. Furthermore, both PKC-*δ* phosphorylation and the total levels of PKC-*δ* were significantly higher after OGD/R; however, C+P annulled this trend at 24 hours of reoxygenation. Similarly, OGD/R facilitated the raise in the p-PKC-*δ*/PKC-*δ* ratio, but C+P decreased this trend at 24 hours of reoxygenation. ^∗^*p* < 0.05, ^∗∗∗^*p* < 0.001 versus the OGD group; ^#^*p* < 0.05, ^###^*p* < 0.001 versus the OGD/C+P group. Results are shown as mean ± SE of three independent experiments.

Since PKC-*δ* phosphorylation influences its catalytic activity [30], we investigated levels of both total and phosphorylated PKC-*δ*. In brain tissue, p-PKC-*δ* concentration was raised at 6 hours of reperfusion (*p* < 0.01). C+P treatment resulted in significantly lowered p-PKC-*δ* compared with I/R groups. Total PKC-*δ* levels increased in the infarcted area at 24 hours of reperfusion (*p* < 0.05). C+P decreased total PKC-*δ* protein levels (*p* < 0.05, *p* < 0.001). Additionally, the p-PKC-*δ*/PKC-*δ* ratio significantly increased post-MCAO (*p* < 0.01); however, the ratio decreased when C+P was administered at 6 hours of reperfusion (*p* < 0.001) (Figure 5(a)). In SHSY5Y cells at 24 hours of reoxygenation, both p-PKC-*δ* and PKC-*δ* levels were elevated after OGD (*p* < 0.05) whereas C+P administration reduced these levels (*p* < 0.05). Also, the p-PKC-*δ*/PKC-*δ* ratio rose post-OGD/R (*p* < 0.05, *p* < 0.001) but was suppressed by C+P treatment at 6 and 24 hours of reoxygenation (*p* < 0.05, *p* < 0.001) (Figure 5(b)).

### 3.5. C+P Treatment Decreased PKC-*δ* Translocation

**Figure 6:**
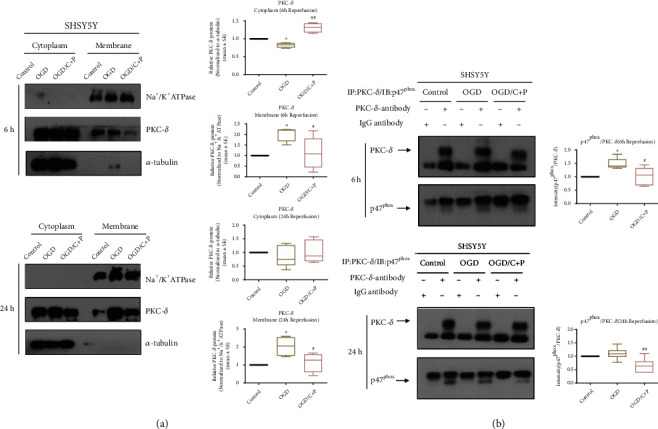
C+P suppressed the translocation of PKC-*δ* and interaction of PKC-*δ* with p47^phox^ induced by OGD/R in SHSY5Y cells. (a) Representative bands of cytosolic and membrane PKC-*δ* identified using Western blot at 6 and 24 hours of reperfusion. Bar graphs show semiquantitative PKC-*δ* levels in the cytosol and membrane determined using band density analysis. Cytosolic PKC-*δ* expression decreased but increased in the membrane in the OGD/R group at 6 hours after reoxygenation as compared to the control group. Administration of C+P negated the changes of OGD/R-induced PKC-*δ* expression. No differences were detected in cytosolic PKC-*δ* expression in the OGD/R group at 24 hours of reoxygenation, but OGD/R increased membranous PKC-*δ* expression as compared to the control group. C+P attenuated the raised cellular membrane PKC-*δ* concentration. ^∗^*p* < 0.05, as compared to the OGD group; ^#^*p* < 0.05, ^##^*p* < 0.01, as compared to the OGD/C+P group. All the quantifications were normalized to *α*-tubulin (cytosol) or Na^+^/K^+^ ATPase (membrane), respectively. Results are shown as mean ± SE of three independent experiments. (b) Representative bands of coimmunoprecipitation of PKC-*δ* and p47^phox^ in SHSY5Y cells, identified via the Western blot technique at 6 and 24 hours of reoxygenation. Bar graphs show the ratios of p47^phox^/PKC-*δ* identified using band density analysis. IgG antibody served as the control. In comparison to IgG control, a significant enhancement in p47^phox^ was detected in the immunoprecipitation complex with the PKC-*δ* antibody from cell lysates, which suggests a heightened interaction of PKC-*δ* and p47^phox^. Moreover, the interaction of PKC-*δ* and p47^phox^ increases after 6 hours of reoxygenation, while C+P treatment reduced the interaction. Although the interaction between the control and the OGD group was not significant at 24 hours of reoxygenation, the interaction did increase, and C+P treatment lowered the interaction between PKC-*δ* and p47^phox^. ^∗^*p* < 0.05 versus the OGD group; ^#^*p* < 0.05, ^##^*p* < 0.01 versus the OGD/C+P group. The data is represented as mean ± SE of three independent experiments.

Western blot analyses were performed to investigate PKC-*δ* cytosolic and membrane expression of SHSY5Y cells, presented in Figure 6(a). In comparison to the control group, cytosolic expression of PKC-*δ* expression decreased (*p* < 0.05) and membrane expression increased (*p* < 0.05) in the OGD/R group at 6 hours after reoxygenation, whereas C+P treatment prevented changes in PKC-*δ* expression caused by OGD/R (*p* < 0.05, *p* < 0.01). Against the OGD/R group at 24 hours of reoxygenation to the control, no differences in PKC-*δ* expression in the cytosol were seen; however, there was significantly increased PKC-*δ* expression in the membrane (*p* < 0.05). C+P lessened increased membrane PKC-*δ* expression (*p* < 0.05).

### 3.6. C+P Treatment Inhibited PKC-*δ* and p47^phox^ Interaction

In order to determine the functional interaction of PKC-*δ* and p47^phox^, whole cell lysates from neuronal SHSYSY cells were immunoprecipitated with PKC-*δ* or IgG antibody followed by antibody treatment against PKC-*δ* and p47^phox^ with Western blot. The IgG antibody served as the control. In comparison to the IgG control, significant increases in p47^phox^ were detected in the immunoprecipitation complex with the PKC-*δ* antibody from cell lysates, indicating an interaction between PKC-*δ* and p47^phox^. Moreover, after 6 hours of reoxygenation, OGD resulted in increased interaction of PKC-*δ* and p47^phox^ (*p* < 0.05), whereas treatment with C+P reduced interaction (*p* < 0.05). There was also an observable, but statistically insignificant increase in the interaction between the control and OGD groups at 24 hours of reoxygenation while C+P treatment attenuated the interaction of PKC-*δ* and p47^phox^ (*p* < 0.01) (Figure 6(b)).

### 3.7. C+P Promoted Increased Expression of MnSOD

**Figure 7:**
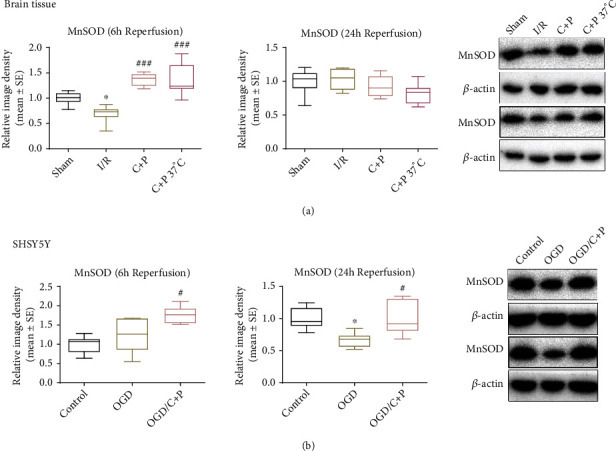
C+P facilitated the expression of MnSOD after cerebral ischemia or OGD/R. (a) Representative bands of MnSOD in brain tissue as identified using Western blot at 6 and 24 hours of reperfusion. *β*-Actin served as an internal control. Semiquantitative levels of MnSOD are displayed in bar graphs, identified using band density analysis. MnSOD protein levels decreased after I/R. C+P administration significantly accelerated MnSOD expression at 6 hours of reperfusion (*n* = 7). ^∗^*p* < 0.05 versus the sham group; ^###^*p* < 0.001 versus the I/R group. The data is presented as mean ± SE. (b) Representative bands of MnSOD expression in SHSY5Y cells as identified using Western blot at 6 and 24 hours of reoxygenation. *β*-Actin protein served as an internal control. Bar graphs show semiquantitative levels of MnSOD as identified using band density analysis. MnSOD protein levels decreased at 24 hours of reoxygenation. However, C+P therapy elevated MnSOD expression at 6 and 24 hours of reoxygenation. ^∗^*p* < 0.05 versus the OGD group; ^#^*p* < 0.05 versus the OGD/C+P group. Results are shown as mean ± SE of three independent experiments.

MnSOD levels were significantly decreased for the 2-hour MCAO/24-hour reperfusion group (*p* < 0.05). However, treatment with C+P significantly reversed these decreased MnSOD levels, regardless of temperature control (*p* < 0.001) (Figure 7(a)). Similarly, in the cell experiments, OGD induced a significant reduction of the MnSOD level at 24 hours of reoxygenation. As hypothesized, C+P administration significantly increased MnSOD expression at both 6 and 24 hours of reoxygenation (*p* < 0.05) (Figure 7(b)).

## 4. Discussion

**Figure 8:**
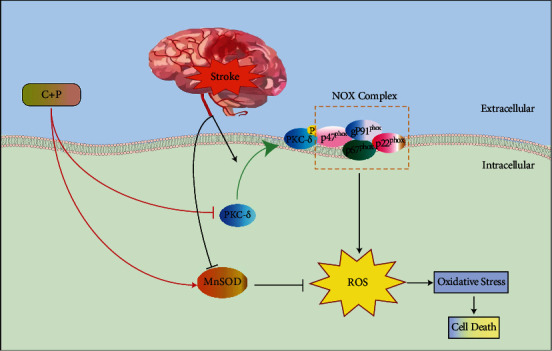
Schematic illustration of neuroprotection of C+P through the PKC-*δ*/NOX/MnSOD pathway. After I/R, PKC-*δ* translocated to the cell membrane is phosphorylated and activated. PKC-*δ* interacted with p47^phox^ which facilitates the activation of NOX. Moreover, MCAO also induced the elevated expression of NOX subunits gp91^phox^, p47^phox^, p67^phox^, and p22^phox^, resulting in NOX activation. NOX activation further increased ROS production and oxidative stress. Additionally, MnSOD expression is repressed after stroke. C+P suppressed both PKC-*δ* and NOX activation and promoted MnSOD expression, resulting in the reduced ROS production and oxidative stress.

This study sought to explore C+P-induced neuroprotection after ischemic stroke. Our findings reinforce findings from previous studies which assert that treatment with C+P reduces neurologic infarction volumes, neurologic deficits, and apoptotic cell death in MCAO rats, which was demonstrated with concurrent use of NOX and PKC-*δ* inhibitors. In addition, we found that C+P also decreased ROS generation, NOX activity, NOX protein subunit expression, and the ratio of p-PKC-*δ*/PKC-*δ*, while increasing levels of MnSOD. NOX activity, NOX subunit protein expression, and ROS also decreased when subjected to NOX and PKC-*δ* inhibitors. Meanwhile, C+P therapy reduced ROS levels, NOX subunit protein expression, p-PKC-*δ*/PKC-*δ*, and increased MnSOD as well as suppressing PKC-*δ* membrane translocation and p47^phox^ and PKC-*δ* interaction in the OGD/R model. Consistent with our previous research, PKC-*δ* and NOX seem to be key regulators by which C+P exerts its neuroprotective effects [5, 12]. This present study further explores the specific mechanism by which C+P inhibits oxidative stress and exerts neuroprotective effects through the PKC-*δ*/NOX/MnSOD pathway following ischemic stroke. This could be due to altered NOX subunit protein expression and PKC-*δ* regulation via phosphorylation, membrane translocation, and changes in the PKC-*δ* and p47^phox^ interaction (Figure 8).

Using ischemic stroke models in a prior study, we found that administration of C+P enhanced neuroprotection by attenuating harmful metabolic cascades. These findings demonstrated C+P's cerebro-protective effects in models of ischemia, demonstrated through infarction volume reductions and decreased neurological deficits after stroke [5]. In addition, C+P's neuroprotective influence in ischemic stroke seems to be in part due to its ability to attenuate hyperglycolysis [9], disruption of the blood-brain barrier [22], inflammatory cascades [31], activation of inflammasomes [32], and apoptotic cell death [12]. C+P can induce therapeutic hypothermia; however, its neuroprotective ability post-stroke appears to be at least partially independent of its hypothermic effects [5, 9, 31, 32]. Recently, we reported that C+P suppressed neuroinflammatory reactions and NLRP3 inflammasome activation even without induction of hypothermia. We found that inhibition of the NLRP inflammasome could be achieved through reduced expression of several vital protein regulators, serving as possible focuses for neuroprotective therapy post-ischemic stroke [25]. In parallel, our current study indicates that C+P's neuroprotective effects after ischemic events are partially attributed to their hypothermic effects. However, C+P's cerebro-protective effects could also result from their effects on components of oxidative stress pathways, such as of PKC-*δ*, ROS, and NOX enzymes. Regardless of temperature control, treatment with C+P was also found to significantly reverse the post-MCAO decrease in MnSOD, a vital central nervous system antioxidant enzyme that strengthens cellular resistance to ROS after cerebral ischemia. Through these mechanisms, C+P therapy was found to significantly reduce infarction volumes, neurological deficits, and apoptotic cell death. C+P's neuroprotective effects seen in temperature-controlled environments reveal that their ability to enhance neuroprotection via pharmacological mechanisms is independent of hypothermia, which was confirmed by cell line experimentation.

ROS are vital components in a rising number of diseases, as they worsen oxidative stress while simultaneously enhancing tissue dysfunction [33]. Oxidative stress is a major factor in neurologic damage resulting from ischemic stroke. Mitochondrial overproduction of ROS is theorized to be the driving force in oxidative stress. NOX enzymes, which transfer electrons across biological membranes, have been shown to be significant ROS generators in cerebral tissue postischemic event [34]. NOX enzymes use NADPH to catalyze molecular oxygen reduction, producing a superoxide anion. NADPH is supplied by the hexose monophosphate shunt for NOX enzymatic activity [35]. NOX metabolic activity worsens I/R injury [15], and inhibition of NOX has been shown to reduce neurologic damage after I/R injury [36]. The NOX complex includes both membrane-bound and cytosolic elements. In its resting state, the NOX catalytic region is composed of gp91^phox^ and p22^phox^, which are membrane-integrated flavocytochromes. The cytosolic elements include p47^phox^ and p67^phox^ [14, 37]. In order to activate NADPH oxidase, there must be membrane assembly of cytosolic p47^phox^, p67^phox^, p22^phox^, and gp91^phox^, resulting in activation of gp91^phox^, the catalytic subunit [14]. It has been reported that I/R decreased p47^phox^/p67^phox^ in the cytosol and increased p47^phox^/p67^phox^/gp91^phox^ in the membrane [38]. Upregulation of all major NOX subunits (p47^phox^, p67^phox^, p22^phox^, and gp91^phox^) has also been found to be associated with increased NOX activity post-stroke [39–42]. Our current study showcased significantly decreased ROS levels, NOX enzyme activity, and levels of p47^phox^, p67^phox^, p22^phox^, and gp91^phox^ proteins after C+P therapy, consistent with the results of the NOX inhibitor group or PKC-*δ* inhibitor group. This furthers indicates a role for C+P in inhibition of oxidative stress through repression of the PKC-*δ*/NOX pathway.

PKC is a known component of upstream signaling in NADPH oxidase activation [38]. Further, chlorpromazine has been found to lower cadmium-induced oxidative renal damage, stemming from its ability to mitigate oxidative stress and stabilize calcium homeostasis via PKC inhibition [43]. Prior studies have shown that administration of C+P reduces cell apoptosis via the NOX-Akt/PKC pathway [12]. PKC is a family of serine-threonine kinases, which control a wide variety of cell functions and have been involved in various diseases, including I/R injury [44–46]. This family is comprised of nine genes which express structurally related phospholipid-dependent kinases with separate regulatory mechanisms and distinct distribution among tissues. Various studies have connected PKC-*δ* to cell death in a variety of cultured cells [47], including neuronal cells [48]. PKC-*δ*-null mice have been shown to have a substantial reduction in stroke size (70%) when assessed against wild-type mice post transient MCAO and subsequent reperfusion therapy [46]. Additionally, NMDA-dependent upregulation of PKC-*δ* around the lesion significantly increased after I/R, along with other proteins known to be involved in neuronal death, like heat-shock proteins and haem oxygenase-1 [49–51]. PKC-*δ* appears to be a substantial part of oxidative stress regulation, likely through NOX stimulation in reperfusion injury [46]. In turn, PKC-*δ* activity is controlled by phosphorylation patterns and subcellular translocation in a context-dependent manner [30]. For example, after stroke, the phosphorylation level of PKC-*δ* increases [52, 53]. This phosphorylated state activates PKC-*δ*, promoting NOX and ROS production [54]. Moreover, post-stroke, the translocation of PKC-*δ* from the cytosol to the membrane is facilitated, which impacts its function [38, 55]. PKC interactions with other proteins are also vital to the functions of PKC itself and the proteins it interacts with [56]. Prior research has demonstrated that ROS production is stimulated by activating the PKC-*δ*-p47^phox^ axis via PKC-*δ* activation, done by facilitating its translocation to the membrane and interacting with p47^phox^ in vascular smooth muscle cells [57]. In our current study, we observed that C+P inhibited ROS production ordinarily produced by the PKC-*δ*-p47^phox^ pathway. It accomplished this by reducing PKC-*δ* phosphorylation, PKC-*δ* membrane translocation, and PKC-*δ* and p47^phox^ interaction.

In mammalian cells, MnSOD is a crucial antioxidant enzyme located in the mitochondria. MnSOD detoxifies superoxide, the major byproduct of mitochondrial respiration [58]. Consequently, MnSOD plays a crucial part in cell defense from oxidative stress [59]. MnSOD is highly expressed in neuronal cells and is imperative for maintaining protection from oxidative stress [60]. MnSOD is therefore a critical endogenous protective mechanism against cerebral ischemic damage. Protein expression of MnSOD was suppressed after reperfusion due to deactivation of the STAT3 gene, which regulates MnSOD expression in neuronal cells under regular conditions [61, 62]. OGD/R induction has also been found to downregulate MnSOD [63]. Moreover, MnSOD-deficient mice were found to have greater infarct volumes and levels of cell death post-ischemic event. Previous studies discerned that loss of MnSOD leads to reduced antioxidant activity and higher levels of uncoupled superoxide anion, which exacerbates ischemic injury and results in expansion of the infarcted penumbral area [64, 65]. Conversely, overexpression of MnSOD provided neuroprotection following cerebral I/R. For example, overexpressors of MnSOD had not increased brain hemorrhage and vascular endothelial cell death rates that were found in underexpressor and knockout models [66]. In our study, administration of C+P increased the expression of MnSOD, which may have further reduced the oxidative stress damage caused by ROS, conferring additional neuroprotection. These findings contribute not only to the growing section of literature exploring neuroprotective benefits of C+P therapy, but also to that of the antioxidant properties of MnSOD.

## 5. Conclusion

C+P dual therapy has evident neuroprotective benefits in the context of ischemic stroke. Several studies suggest that its benefits are conveyed through pharmacologically induced hypothermia. However, others have substantiated its benefits in temperature-controlled settings, thereby imploring the discovery of additional mechanisms for its neuroprotection. Our study sought to further explore these mechanisms and found that C+P-mediated antioxidant defense is through the PKC-*δ*/NOX/MnSOD pathway following acute ischemic stroke. The findings of this study provide valuable insight for future patients who suffer from strokes, as therapies targeting these pathways may significantly enhance neuroprotection. Future studies should focus on exploring these neuroprotective mechanisms in greater depth.

## Data Availability

The data used to support the findings in this study are available from the corresponding author upon reasonable request.
